# Emerging Markers of Atherosclerosis Before and After Bariatric Surgery

**DOI:** 10.1007/s11695-014-1407-7

**Published:** 2014-09-28

**Authors:** Justyna Domienik-Karłowicz, Zuzanna Rymarczyk, Olga Dzikowska-Diduch, Wojciech Lisik, Andrzej Chmura, Urszula Demkow, Piotr Pruszczyk

**Affiliations:** 1 Department of General Medicine and Cardiology, Medical University of Warsaw, Lindley’a 5, 02-005 Warsaw, Poland; 2 Department of General Surgery and Transplantology, Medical University of Warsaw, Nowogrodzka 59, 02-005 Warsaw, Poland; 3 Department of Laboratory Medicine and Clinical Immunology of Developmental Age, Medical University of Warsaw, Marszałkowska 24, 00-576 Warsaw, Poland

**Keywords:** Atheroslerosis, Bariatric sugery, Morbid obesity, Emerging markers, MMP

## Abstract

**Objective:**

The objective of this study was to assess the emerging biochemical markers of arterial remodeling in patients with morbid obesity before and after surgical treatment and to compare the results to a control group.

**Material and Methods:**

The prospective study included 40 patients with BMI 47.73 ± 6.18 kg/m^2^, qualified for elective bariatric surgery and re-examined 6 months after the surgery. The control group consisted of non obese, age and sex matched 15 subjects. Following laboratory examinations were performed in all patients: basic laboratory examinations, MMP-2, MMP-9, adiponectin, PAI-1, CD40L, E-selectin.

**Results:**

Examination of patients 6 m after bariatric surgery revealed a 34.57 ± 9.71 reduction in excess body weight. Comparison of the study group at two time points revealed differences in adiponectin, MMP-2 and MMP-9 levels. Hypoadiponectinemia was observed in 35 % patients 6 months after bariatric surgery compared to 90 % patients before the surgery. In addition, a strong correlation was observed between body fat mass and adiponectin levels (*r* = −0.504, *p* = 0.055). Moderate correlations were demonstrated between E-selectin levels and BMI (*r* = 0.361; *p* = 0.022), and metalloproteinase-9 levels (*r* = 0.326; *p* = 0.040). In addition, strong relationship was demonstrated between MMP-2 and MMP-9 (*r* = 0.502; *p* = 0.001), and moderate between MMP-2 and adiponectin levels (*r* = 0.449; *p* = 0.003). MMP-9 levels were moderately correlated with HDL-cholesterol levels (*r* = 0.316; *p* = 0.046).

**Conclusions:**

Assessment of laboratory markers of arterial remodeling and metabolism suggest their adverse changes in patients with morbid obesity. However, body mass reduction due to bariatric surgery decreases inflammatory status, improves biochemical markers of arterial remodeling as well as to beneficial changes in the metabolism.

Obesity is an established risk factor for a number of cardiovascular disorders. The adipose tissue is a complex, metabolically active organ and a source of pro-inflammatory cytokines. Hormones produced by the adipose tissue play a key role in the energy balance and the metabolism of carbohydrates and lipids [1]. Due to the activity of pro-inflammatory cytokines, individuals with excess adipose tissue experience chronic inflammation which leads to progressive dysfunction in the endothelium, recruitment of further inflammatory cells, remodeling of the blood vessels [2–4] with the development of intermediate plaque. Activation of inflammatory cells producing cytokines and chemokines leads to the development of complex plaque [3].

Development of atherosclerosis involves activity of extracellular matrix metalloproteinases (MMP-2 and MMP-9), adiponectin, E-selectin, PAI-1, and CD40L. Extracellular matrix metalloproteinases are involved in remodeling of extracellular matrix and basal membranes. Disturbed equilibrium between MMPs and their inhibitors (tissue inhibitors of metalloproteinases, TIMPs) [4] and α-2-macroglobulin leads to enhanced proteolysis and endothelial damage or to accumulation of matrix constituents [5]. MMPs were demonstrated to play an important role in the atherosclerotic process, modulation of adipogenesis, [6] remodeling of the walls of blood vessels and myocardium [7, 8].

Adiponectin is a polypeptide hormone, produced and secreted into blood only by adipose tissue cells [9]. According to many authors, production of adiponectin is, in contrast to other hormones originating from the adipose tissue, negatively correlated with the body weight, visceral fat, and BMI, [10] although not all studies have confirmed this hypothesis [11–14]. Abbasi et al. demonstrated that a small drop in body weight due to non-surgical methods does not significantly affect the adiponectin levels; [13] In clinical practice, hypoadiponectinemia is considered to be a predictor of type II diabetes, lipid disorders, arterial hypertension and non-alcoholic liver fatty liver disease. Schondorf et al. determined the reference adiponectin levels to be in the range of 7–12 mg/L. Values lower than 7 suggest hypoadiponectinemia and are a strong indicator of complications within the cardiovascular system [15, 16].

The CD40 ligand (CD40L) belongs to the family of tumor necrosis factor (TNF alpha) cytokines. It is an element of the CD40/CD40L transmitter system. It should be highlighted that studies published to date suggest elevated sCD40L levels in type II diabetes, [17–19], metabolic syndrome, [17, 20]. Data on the relationship between CD40L levels and obesity and morbid obesity are ambiguous [21, 22].

E-selectin, is a membrane protein from the selectin group. Data on the relationship between E-selectin levels and body weight/BMI are ambiguous. Palomo et al. demonstrated no difference between E-selectin levels in a group of metabolic syndrome patients and patients without metabolic syndrome [20]. Hanusch-Enserer et al., who conducted a study in a group of 32 patients with morbid obesity subjected to LAGB demonstrated that E-selectin levels were positively correlated with BMI both before and after the surgery [23].

Elevated plasminogen activator inhibitor-1 (PAI-1) levels, were observed in patients with obesity. It was suggested that bariatric surgeries lead to positive outcomes, as PAI-1 levels in patients after the surgery are lower than before the surgery [24].

## Objectives

The objective of this study was to assess the emerging biochemical markers of arterial remodeling in patients with morbid obesity before and after gastric bypass surgery and to compare the results to a control group consisting of individuals with normal body weight.

## Methods

The prospective study included 40 female patients with morbid obesity, BMI 47.73 ± 6.18 kg/m^2^, qualified for elective gastric bypass surgery and re-examined 6 months after the surgery. Patients with the history of myocardial infarction or with significant valvular heart disease, chronic renal disease (GFR < 60 ml/min/1,73 m^2^), chronic hepatic insufficiency, or chronic obstructive pulmonary disease, smokers (>20 cigarettes/day), and patients with obstructive sleep apnea (acute, AHI ≥ 30) were excluded from the study.

The control group consisted of non obese, age and sex matched 15 subjects.. Anthropometric measurements and laboratory analyses were performed in all study subjects. Body weight, fat body mass and lean body mass values were determined using Tanita TBF-300 Body Composition Analyser based on electric bioimpedance measurements. Ideal body weight (IBW) was calculated according to the following formula: (age in cm–100)–10 %; excess body weight (EBW) was calculated according to the following formula: current body weight–ideal body weight; % excess weight loss (%EWL) was calculated according to the following formula: (body weight before surgery–body weight 6 months after surgery)/ excess body weight before surgery x 100.

Following laboratory examinations were performed in all patients: complete blood counts, lipid profile, creatinine (mg/dL), hsCRP (mg/L), HbA_1_C (%), serum glucose (mg/dl) and serum insulin (μIU/mL) by immunochromatography, MMP-2 – Ray Biotech ELISA kit, MMP-9 – Ray Biotech ELISA kit, adiponectin – Teco Medical ELISA kit, PAI-1 – Biovendor ELISA kit, CD40L – GEN PROBE diaclone ELISA kit, E-selectin – GEN PROBE diaclone ELISA kit. The study protocol was approved on 31 March 2009 by the Local Bioethics Committee.

### Statistical Analysis

Quantitative variables were analyzed using standard descriptive statistic measures: means, standard deviations for normal distribution variables and medians and ranges for non-normal distribution variables. The hypothesis of variable distribution being consistent with normal distribution was tested for quantitative variables. To this end, Shapiro-Wilk’s test and Q-Q plot test were used. When distribution of a particular variable was normal, the hypothesis of the means in two study groups being equal was examined using the Student’s *t*-test. Wilcoxon’s test for independent samples was used to compare two groups in case of parameters demonstrating deviations from normal distribution. The analysis of quantitative parameters of management involved the following dependent sample tests: Student’s *t*-test for normal distribution variables and Wilcoxon’s test for non-normal distribution variables. Correlations between quantitative variables involved Pearson’s coefficient for normal distribution variables and Spearman’s coefficient for non-normal distribution variables. The relationships between qualitative variables were studied in contingency tables using the chi-square test or Fisher’s exact test when the expected values in table cells were not high enough (i.e. above 5). The analysis of changes in qualitative parameters during the treatment involved the McNemar’s test for the symmetry of related samples. The level of statistical significance was established at *p* < 0.05. Calculations were performed using the SAS 9.2 software.

## Results

### Overall Clinical Characteristics of Morbid Obesity Patients

A total of 40 patients with pathological obesity, aged 36.4 ± 9.0 years were included into the study. All patients were re-examined approximately 6 month after the surgery Table 1 presents the anthropometric parameters of patients qualified for the surgery and patients in the control group.

**Table 1:** Anthropometric parameters of patients qualified for bariatric surgery (OB1) and the control group (CG)

	OB1 (*n* = 40)	CG (*n* = 15)	p
Body weight (kg)	132.03 ± 18.42	60.73 ± 5.12	<0.0001
BMI (kg/m^2^)	47.73 ± 6.18	21.61 ± 1.41	<0.0001
FAT (%)	49.15 ± 3.83	25.13 ± 4.88	<0.0001
FFM (kg)	66.78 ± 7.84	45.37 ± 3.7	<0.0001
BSA M (m^2^)	2.47 ± 0.20	1.68 ± 0.09	<0.0001
EBW (kg)	72.34 ± 16.75		

Arterial hypertension was diagnosed in 38 (95 %) patients. Initially 30 (75 %) patients received 2 or more hypotensive drugs (including ACE inhibitors and a diuretic); while 8 (20 %) patients received only one hypotensive drug (ACE inhibitor). Pre-diabetes was diagnosed in 11 (27.5 %) patients, including impaired fasting glucose in 6 patients (15 %) and impaired glucose tolerance in 7 (17.5 %) patients [25]. Both of these abnormalities were observed simultaneously in 2 patients (5 %). Patients diagnosed with type 2 diabetes were excluded from the study. Lipid metabolism disorders were observed in 22 (55 %) patients, while 8 (20 %) patients were diagnosed with obstructive sleep apnea of not more than moderate intensity.

### Assessment of Blood Chemistry Parameters in Patients with Morbid Obesity

Table 2 presents the blood chemistry parameters observed in patients with morbid obesity

**Table 2:** Assessment of blood chemistry parameters in patients with morbid obesity

	mean	±SD	median	range
fasting glucose (mg/dL)	90.4	10.19	89	67.0–124.0
fasting insulin (μIU/mL)	17.68	11.29	14.07	5.45–56.33
HOMA-IR	3.89	2.33	3.31	1.14–11.68
creatinine (mg/dL)	0.76	0.16	0.7	0.5–1.3
total cholesterol (mg/dL)	199.15	35.13	197.5	129.0–296.0
HDL-chol (mg/dL)	52.55	10.97	50.5	36.0–86.0
LDL-chol (mg/dL)	120.3	31.59	119.5	52.0–240.0
triglycerides (mg/dL)	124.78	47.7	114	31.0–255.0
hsCRP (mg/L)	10.19	6.92	8.8	1.9–32.02
HbA_1_C (%)	5.8	0.89	5.65	5.2–6.6
CD40L (ng/mL)	3.63	34.3	1.96	0.02–17.51
E-selectin (ng/mL)	68.49	38.94	56.48	21.87–191.71
Adiponectin (ng/mL)	4,976.34	2,728.36	4,345.94	1,893.15–16,270.60
MMP-2 (ng/mL)	70.15	74.11	46.19	11.23–419.06
MMP-9 (ng/mL)	10,061	4,510.67	8,546.49	2,210.59–30,522.40
PAI (pg/mL)	257,373	175,597	246,291	20,639.20–818,896

### Comparison of Blood Chemistry Parameters in the Group of Patients with Morbid Obesity (OB1) and the Control Group (CG)

Table 3 presents comparison of blood chemistry parameters in the group of patients with morbid obesity and the control group

**Table 3:** Comparison of blood chemistry parameters in the group of patients with morbid obesity and the control group

	OB1 (median, range)	CG (median, range)	p
fasting glucose (mg/dL)	89; 67–124	90; 81–102	NS
fasting insulin (μIU/mL)	14.07; 5.45–56.33	6.81; 3.57–12.73	<0.0001
HOMA-IR	3.31; 1.14–11.68	1.61; 0.78–3.02	<0.0001
creatinine (mg/dL)	0.7; 0.5–1.3	0.8; 0.6–0.9	NS
total cholesterol (mg/dL)	197.5; 129.0–296.0	183; 129–233	NS
HDL-chol (mg/dL)	50.5; 36.0–86.0	69; 50–85	<0.0001
LDL-chol (mg/dL)	119.5; 52.0–240.0	105; 52–145	0.04
triglycerides (mg/dL)	114; 31.0–255.0	51; 30–203	0.001
hsCRP (mg/L)	8.8; 1.9–32.02	0.60; 0.1–2.40	NS
HbA_1_C (%)	5.65; 5.2–6.6	5.60; 5.30–5.80	0.001
CD40L (ng/mL)	1.96; 0.02–17.51	0.56; 0.06–1.77	0.0008
E-selectin (ng/mL)	56.48; 21.87–191.71	54.41; 20.73–110.01	NS
Adiponectin (ng/mL)	4,345.94; 1,893.15–16,270.60	14,548.31; 5,392.68–26,099.29	<0.0001
MMP-2 (ng/mL)	46.19; 11.23–419.06	28.05; 4.31–295.86	0.03
MMP-9 (ng/mL)	8,546.49; 2,210.59–30,522.40	7,304.87; 7,011.3–7,660.98	<0.0001
PAI (pg/mL)	246,291; 20,639.20–818,896	84,523.52; 33,281.73–181,843.26	0.0003

### Assessment Pre- and Post-Surgery Anthropometric Parameters in Patients with Morbid Obesity

Table 4 presents the comparison of anthropometric parameters in the study group before the bariatric surgery and 6 months after bariatric surgery.

**Table 4:** Comparison of anthropometric parameters of patients 6 months after bariatric surgery (*n* = 40)

	OB1	OB2	p
Body weight (kg)	132.03 ± 18.42	97.46 ± 15.35	<0.0001
BMI (kg/m^2^)	47.73 ± 6.18	35.22 ± 5.20	<0.0001
FAT (%)	49.15 ± 3.83	38.20 ± 5.66	<0.0001
FFM (kg)	66.78 ± 7.84	59.90 ± 8.92	<0.0001
BSA M (m^2^)	2.46 ± 0.19	2.12 ± 0.19	<0.0001
EBW (kg)	72.34 ± 16.75	37.77 ± 13.97	<0.0001

Table 5 compares the study results comparing blood chemistry parameters in patients before bariatric surgery and 6 months after bariatric surgery.

**Table 5:** Comparison of blood chemistry parameters in patients before bariatric surgery and 6 months after bariatric surgery

	OB1 (median, range)	OB2 (median, range)	p
fasting glucose (mg/dL)	89; 67–124	85.00; 66.00–109.00	0.0139
fasting insulin (μIU/mL)	14.07; 5.45–56.33	7.72; 1.67–108.00	<0.0001
HOMA-IR	3.31; 1.14–11.68	1.76; 0.35–24.80	<0.0001
creatinine (mg/dL)	0.7; 0.5–1.3	0.80; 0.5–1.0	NS
total cholesterol (mg/dL)	197.5; 129.0–296.0	191.00; 122.00–257.00	<0.0001
HDL-chol (mg/dL)	50.5; 36.0–86.0	55.00; 38.00–80.00	NS
LDL-chol (mg/dL)	119.5; 52.0–240.0	112.50; 57.00–183.00	NS
triglycerides (mg/dL)	114; 31.0–255.0	88.50; 39.00–235.00	<0.0001
hsCRP (mg/L)	8.8; 1.9–32.02	2.80; 0.4–25.50	<0.0001
HbA_1_C (%)	5.65; 5.2–6.6	5.35; 4.70–6.10	<0.0001
CD40L (ng/mL)	1.96; 0.02–17.51	2.00; 0.02–13.95	NS
E-selectin (ng/mL)	56.48; 21.87–191.71	54.05; 14.46–239.31	NS
Adiponectin (ng/mL)	4,345.94; 1,893.15–16,270.60	8,972.87; 2,940.06–26,711.11	<0.0001
MMP-2 (ng/mL)	46.19; 11.23–419.06	39.26; 8.90–141.47	0.007
MMP-9 (ng/mL)	8,546.49; 2,210.59–30,522.40	8,024.56; 2,210.59–46,160	0.039
PAI (pg/mL)	246,291; 20,639.20–818,896	167,240.43; 19,175.87–622,270.64	NS

In addition, a strong negative correlation was observed between body fat mass and adiponectin levels (*r* = −0.504, *p* = 0.055).

Moderate correlations were found between E-selectin levels and BMI (*r* = 0.361; *p* = 0.022), MMP-9 levels (*r* = 0.326; *p* = 0.040). Mutual strong correlation was observed between metalloproteinases MMP-2 and MMP-9 (*r* = 0.502; *p* = 0.001). In addition, (“strong” removed) moderate relationship was demonstrated between MMP-2 and adiponectin levels (*r* = 0.449; *p* = 0.003). On the other hand, MMP-9 levels were moderately correlated, as mentioned previously, with E-selectin levels (*r* = 0.326; *p* = 0.040) as well as with HDL-cholesterol levels (*r* = 0.316; *p* = 0.046).

### Changes in Studied Parameters 6 Months After Bariatric Surgery

Examination of patients 6 months after bariatric surgery revealed a 34.57 ± 9.71 kg reduction in excess body weight. This change was accompanied by changes in laboratory parameters. Statistically significant changes and changes important for presentation of the results are presented in Tables 6 and 7.

**Table 6:** Correlations of changes in CD40L levels following bariatric surgery compared to baseline levels (Δ E-CD40L) with changes in levels of selected biochemical parameters related to endothelial function

Parameter: Δ CD40L	r	p
Δ total cholesterol	0.937	<0.0001
Δ LDL-cholesterol	0.854	<0.0001
Δ HDL-cholesterol	0.477	0.0018
Δ PAI	0.431	0.0097
Δ E-selectin	0.4036	0.012

**Table 7:** Correlations of changes in E-selectin levels following bariatric surgery compared to baseline levels (Δ E-selectin) with changes in levels of selected biochemical parameters related to endothelial function

Parameter: Δ E-selectin	r	p
Δ total cholesterol	0.348	0.0277
Δ CD40L	0.854	<0.0001
Δ MMP-9	0.302	0.0587

## Discussion

The study assessed, in a non-invasive manner, emerging markers of atherosclerosis in patients with morbid obesity as compared to the control group with normal body weight, as well as the efficacy of gastric bypass surgery of obesity in normalization of these parameters. A total of 40 female patients with morbid obesity, meeting the qualification criteria for bariatric surgery, were qualified for the study [26]. Patients qualified for gastric bypass surgery were not different from the control group subjects in age while differing in BMI range for obvious reasons. When comparing both groups, obese patients were found to exhibit higher fasting insulin levels and HOMA index values. In 19 patients with morbid obesity (48,71 %) fasting insulin levels exceeding 15μIU/mL and suggesting hyperinsulinemia. At the same time, insulin resistance was diagnosed in 67 % of morbidly obesity patients. No cases of hyperinsulinemia or insulin resistance were observed in the control group.

Also according to published reports mean triglyceride and LDL-cholesterol levels in the group of patients qualified for the surgery was higher than in the CG while the mean HDL-cholesterol level was lower than in the CG. Medians of CD40L, adiponectin, MMP-2, MMP-9, and PAI-1 levels were higher in the group of morbid obesity patients as compared to the control group [27–29]. Hypoadinectinemia was diagnosed in 90 % of patients qualified for the bariatric surgery and in 20 % patients in the control group. This confirms previous observations suggesting high incidence of lipid metabolism disorders in obese individuals [30, 31].

After the surgery, following a 6-month follow-up period, a significant drop was observed in BMI, from 47.73 ± 6.18 kg/m^2^ to 35.22 ± 5.20 kg/m^2^ (p < 0.0001), and EBW from 72.34 ± 16.75 kg to 37.77 ± 13.97 kg (p < 0.0001). Significant improvement in carbohydrate metabolic parameters was observed following the bariatric surgery. Elevated fasting blood glucose levels were observed in 6 (15 %) patients in the OB1 group; elevated fasting insulin levels of more than 15 μIU/mL were initially observed in 19 (48.7 %) of study group patients. A statistically insignificant drop was observed in the fasting blood glucose levels (90.40 ± 10.19 mg/dL vs. 85.35 ± 9.50 mg/dL; NS), highly significant (p < 0.0001). drop in fasting insulin levels was observed; after the follow-up period of 6 months, fasting hyperinsulinemia was diagnosed only in 6 (15 %) patients. A decrease in insulin resistance as measured by the HOMA-IR index was also observed after the surgery. Our observations regarding the disturbed carbohydrate metabolism were in line with those in the available literature. Also Krebs et al., when studying a group of morbid obesity patients without concomitant type II diabetes over a similar follow up period observed no significant changes in fasting blood glucose levels and a statistically significant drop in fasting insulin levels [32]. Increase in adiponectin levels correlated with the drop in body weight as the result of bariatric surgery was demonstrated in a number of studies. In our study material, hypoadiponectinemia, defined as adiponectin levels of <7,000 ng/mL was observed in 90 % of patients before the surgery and 35 % of patients after the 6-month follow-up period. We believe that this change is worth a particular mention as, according to observations by Schondorf et al., adiponectin levels of less than 7,000 ng/mL are a predictor of very high risk of cardiovascular complications [15]. In this study, the change in adiponectin levels was strongly correlated with the change in hsCRP levels (*r* = −0.519, *p* = 0.0008). Therefore, what is very important, the inverse relationship between adiponectin and inflammation expressed as elevated hsCRP levels as described in numerous studies was confirmed [33].

A number of studies demonstrated that MMP-9 levels were higher in patients with obesity and in patients with metabolic syndrome as compared to patients with normal body weight [27, 28]. Kosmala et al. observed that MMP-9 levels were strictly correlated with BMI values in obese patients, [34], while. Derosa et al., Unal et al. and other researches demonstrated the relationship between MMP-9 and body weight parameters in patients with morbid obesity before bariatric surgery [27, 35, 36] as well as after significant body weight reduction as a result of bariatric surgery [37–40]. However, no statistically significant correlation between MMP levels and body weight parameters was observed in this study. It is also worth mentioning that a statistically significant drop in MMP-9 levels was observed along with the reduction in body weight (8546.49; 2210.59–30522.40 ng/mL vs. 8024.56; 2210.59–46,160 ng/mL; *p* = 0.039).

In a study of 328 subjects (163 morbid obesity patients and 165 control group subjects), Derosa et al. observed that the activity of MMP-2 was significantly higher in morbid obesity patients, similar as in the case of MMP-9 [27]. The present study also demonstrated that the blood levels of MMP-2 in OB1 group was statistically significantly higher than in the OB2 group (46.19; 11.23–419.06 ng/mL vs. 39.26; 8.90–141.47 ng/mL; *p* = 0.007) and in the control group. Therefore, it is worth underline lower probability of plaque destabilization, after surgery.

Unek et al. examined the relationship between hsCRP and sCD40L levels and the body weight in a group of 148 patients not suffering from type 2 diabetes. They observed higher hsCRP and sCD40L levels in obese patients as compared to overweight or normal weight patients while also demonstrating a positive correlation between hsCRP and sCD40L levels and BMI in the entire study population (*r* = 0.514, *p* = 0.0001 and *r* = 0.283, *p* = 0.0001, respectively). Guldiken et al. [22] were unable to demonstrate such correlation between sCD40L levels and the BMI as well as between the levels of hsCRP and sCD40L. In our study group, no statistically significant correlation was observed between the levels of sCD40L and hsCRP, as well as between changes of these levels in the follow-up period. However sCD40L levels currently observed in the OB1 group were similar to those observed by Schernthaner et al. in the group of patients with morbid obesity (3.63 ± 4.30 vs. 3.7 ± 1.5 ng/mL) [41]. One should not overlook highlighting the observed strong correlation between sCD40L and PAI-1 (*r* = 0.53115, *p* = 0.0005) as well as moderate correlation between ∆CD40L and ∆PAI-1 (*r* = 0.43109; *p* = 0.0097) in the group of morbid obesity patients, which may contribute to less complications of atherosclerosis, by antypromoting thrombus formation in ruptured plaque. According to the available literature, no correlations between the tested parameters have been studied to date in morbid obesity patients. Hanusch-Enserer et al., who conducted a study in a similar group of patients, observed a statistically significant drop in PAI-1 levels upon follow-up; an insignificant drop was also observed in the study group in the present study [23].

In a study of 34 patients subjected to bariatric surgery, Hanusch-Enserer et al. observed a drop in CD62E (E-selectin) levels during a 6-month follow-up period [23]. In our study group, an insignificant drop in this parameter was observed (56.48; 21.87–191.71 ng/mL vs. 54.05; 14.46–239.21 ng/mL).

## Conclusions

Assessment of laboratory markers of arterial remodeling and metabolism suggest their adverse changes in patients with morbid obesity, which potentially increase cardiovascular risk. However, body mass reduction due to bariatric surgery improves biochemical markers of arterial remodeling parameters as well as to beneficial changes in the metabolism.
